# Editorial: Successful teacher: personality and other correlates

**DOI:** 10.3389/fpsyg.2023.1293759

**Published:** 2023-10-09

**Authors:** Elena Mirela Samfira, Tiberiu Sandu Dughi, Jesús de la Fuente

**Affiliations:** ^1^Teacher Training Department, University of Life Sciences “King Mihai I” from Timisoara, Timisoara, Romania; ^2^Department of Pedagogy, Psychology, and Social Work, “Aurel Vlaicu” University of Arad, Arad, Romania; ^3^Department of Theory and Methods of Educational and Psychological Research, University of Navarra, Pamplona, Spain

**Keywords:** personality traits, teachers, academic success, higher education, teaching, classroom climate

Successful teachers are “builders” of destinies, not just people who come to school to share information with students; they are the ones who are a strong influence on the student's development. They know that have chosen this career for the crucial role of the teaching profession in society, whether society recognizes it or not! But what does such a teacher look like? We believe that above all—beyond the subject they teach—a successful teacher makes students believe in themselves, love school, and have deep values that will channel students' destinies in the future. The personality of the teacher greatly influences the students and can have great consequences many years after the end of school.

The importance of this issue started around the 1920s (e.g., Brubacher, 1921) to outline the characteristics of successful and less successful teachers, with more than 1,000 articles published in this field until nowadays (e.g., Burić et al., 2023; Zhi and Wang, 2023). Thus, this Research Topic offered a professional space for new findings on how teachers' personalities can influence teaching, learning, and classroom climate. The relevance of the concept is still high and 14 authors from six countries and three different continents (Asia, Europe, and South America) have contributed with their articles to build a valuable Research Topic.

Thus, Zhu et al. investigated the mediating effect of substitute teachers' psychological capital on the relationship between organizational commitment and turnover intention. The authors worked with 384 substitute teachers and their results revealed that psychological capital partially mediates the organizational commitment and turnover intention relationship. These results highlighted the importance of teachers' high organizational commitment, to become, more confident in planning their steps and more motivated in achieving goals, even when they are facing challenging experiences. Commitment in the educational area seems to be an essential pillar, strongly related to teachers' burnout or turnover. In this sense, Chen et al. investigated the relationship between social support and professional commitment and the mediating effect of teachers' subjective wellbeing (satisfaction with life and positive affect). Their study was conducted with 778 kindergarten teachers. The results showed that social support does not predict professional commitment directly but indirectly through the mediation of positive affect and life satisfaction (components of subjective wellbeing). The authors highlighted that their model could be a possible way to increase teachers' love for their job and dedication. The motivation behind wellbeing and teachers' retention remains a subject open to investigation. Thus, Shi et al. explored how teachers' motivation related to work engagement, workplace wellbeing, and retention intention. The authors conducted their research working with 1,199 kindergarten teachers. The results revealed three motivation profiles (“low value-high cost,” “moderate all,” and “high-value-low cost”), with teachers with lower education levels being more motivated at work and teachers with higher education levels being more inclined to turnover their profession.

Next, Macovei et al. examined to what extent personality traits, role ambiguity, and relational competence predict teacher subjective wellbeing. The authors worked with 105 university teachers and the results revealed that three of the personality traits, emotionality, extraversion, and conscientiousness were significant predictors of teaching efficacy, school connectedness, and teacher subjective wellbeing. Instead, honesty-humility, agreeableness, and openness to experience were not. These findings highlighted the necessity for universities to have more information about how teachers perceive their roles as university teachers. Based on this, different trainings could be applied to reduce role ambiguity. Next, Yue et al. focused on the influence of teachers' personality characteristics which contribute to the development of occupational anxiety caused by China's “double reduction” policy. Based on the grounded theory, the authors conducted in-depth interviews with 45 in-service teachers. Results indicated that teachers had different understandings of the “double reduction” policy and the level of anxiety was influenced mainly by their subjective understanding of the educational reform, not by external factors. The role of internal and external factors influencing teachers' behaviors was also, approached by Markelj et al. who examined the dynamics of teacher burnout, over the school year, with individual and environmental factors in the school context. The authors worked with 718 teachers, in three waves. The results revealed that teachers experience stress, especially in work not directly related to teaching and that time and energy demands of working with students, teacher characteristics, and classroom management were common significant predictors for teacher burnout, at all three time points.

School context was also taken into consideration by Zhao and Jin who explored the cross-cultural differences (American, Finnish, and Chinese teachers) in teacher perceptions of school climate. Latent class analysis was conducted for each country and gender and teaching experience were used as predictors. The results identified four groups/classes labeled positive participation and teacher-student (TS) relationships, positive discipline and TS relationships, moderate, and low participation. These findings emphasized the necessity to consider cultural differences when drawing on the experiences of other countries. Next, Rad et al. investigated Integrative-Qualitative Intentional Behavior by applying the theory of planned behavior. The research was conducted with 300 preschool teachers. The results revealed that two dimensions namely perceived power/control beliefs and behavioral intention were identified as being the most important ones, in adopting qualitative and inclusive behaviors.

A special category of teachers is English as a foreign language teachers (EFL teachers) and Yan et al. investigated the relationship between EFL teachers' beliefs about classroom-based assessment (CBA) and their assessment practices. They conducted their investigation with 195 Chinese primary school EFL teachers. Their findings presented that teachers' beliefs about the CBA process (planning assessment, collecting learning evidence, making professional judgments, and providing appropriate feedback) were significant predictors for their assessment practices. The authors also emphasized the positive impact of CBA on developing learners' autonomy. Next, Liu et al. focused on EFL teachers' emotions and investigated the relationship between teachers' growth mindset, teaching enjoyment, work engagement, and teacher grit. The research was conducted with 486 Chinese EFL teachers. Their findings, applying structural equation modeling (SEM), revealed that teaching enjoyment, teacher grit, and growth mindset directly predicted EFL teachers' work engagement. Additionally, teaching enjoyment and growth mindset predict indirectly teacher's work engagement through the mediation of teacher grit.

Research in the field of EFL teacher work engagement continues with the study of Heng and Chu, who analyzed other predictors, thus expanding knowledge. The authors investigated the role of self-efficacy, reflection, and resilience as predictors of teachers' work engagement. They worked with 512 EFL teachers and the results showed that teacher work engagement was directly predicted by analyzed variables such as teacher self-efficacy, reflection, and resilience. Teacher reflection also had an indirect impact on work engagement through teacher resilience. Furthermore, teacher reflection influenced work engagement indirectly mediated by resilience and similarly, self-efficacy influenced work engagement indirectly through teacher reflection and resilience. These results could have significant effects on teaching staff, teacher training programs, school administrators, and policymakers, for future EFL teacher instruction and their retention in the EFL educational system and preventing burnout. The EFL teachers' self-efficacy was next investigated by Chen who added the loving pedagogy disposition construct, both variables being considered as protective factors against teachers' burnout. The author worked with 428 English teachers from China and after applying the structural model, he identified the negative effect of loving pedagogy on teacher burnout and the mediating role of teacher self-efficacy. The important role of teacher self-efficacy was highlighted also by Zeng et al. in their meta-analysis by introducing a more complex concept such as information technology integration self-efficacy to be analyzed correlated with technology pedagogical and content knowledge. The authors included in their meta-analysis 28 independent samples, from 2007 to 2022, with a total of 7,777 subjects, and the results indicated that teachers' information technology integration self-efficacy and TPACK have a moderate positive correlation. The relationship between these two variables was moderated by the subjects' career stages, but not by gender, disciplines, teaching stages, and measurement tools. This result highlights the crucial role of integration technology information in the field of teacher education.

The research carried out within the present Research Topic reflects the multitude and complexity of the correlates associated with the teacher's personality. These results could help teachers to become more efficient and flexible in the educational process, aspects that can contribute to their wellbeing. Also, researchers in the educational field could find new Research Topics.

## Author contributions

ES: Conceptualization, Writing—original draft. TSD: Supervision, Writing—review and editing. JF: Supervision, Writing—review and editing.
